# Classification of invasive bloodstream infections and *Plasmodium falciparum* malaria using autoantibodies as biomarkers

**DOI:** 10.1038/s41598-020-78155-y

**Published:** 2020-12-03

**Authors:** Ralf Krumkamp, Nicole Sunaina Struck, Eva Lorenz, Marlow Zimmermann, Kennedy Gyau Boahen, Nimako Sarpong, Ellis Owusu-Dabo, Gi Deok Pak, Hyon Jin Jeon, Florian Marks, Thomas Jacobs, Jürgen May, Daniel Eibach

**Affiliations:** 1 Department of Infectious Disease Epidemiology, Bernhard Nocht Institute for Tropical Medicine, Bernhard Nocht Str. 74, 20359 Hamburg, Germany; 2 German Centre for Infection Research (DZIF), Hamburg-Lübeck-Borstel-Riems, Hamburg, Germany; 3 Department of Infectious Disease Epidemiology, Kumasi Centre for Collaborative Research in Tropical Medicine (KCCR), Kumasi, Ghana; 4 School of Public Health, Kwame Nkrumah University of Science and Technology (KNUST), Kumasi, Ghana; 5 Epidemiology Unit, International Vaccine Institute (IVI), Seoul, Republic of Korea; 6 The Department of Medicine, the University of Cambridge, Cambridge, UK; 7 Research Group Protozoa Immunology, Bernhard Nocht Institute for Tropical Medicine, Hamburg, Germany; 8 First Medical Clinic and Polyclinic, University Medical Center Hamburg-Eppendorf, Hamburg, Germany

**Keywords:** Diagnostic markers, Machine learning, Malaria, Bacterial infection

## Abstract

A better understanding of disease-specific biomarker profiles during acute infections could guide the development of innovative diagnostic methods to differentiate between malaria and alternative causes of fever. We investigated autoantibody (AAb) profiles in febrile children (≤ 5 years) admitted to a hospital in rural Ghana. Serum samples from 30 children with a bacterial bloodstream infection and 35 children with *Plasmodium falciparum* malaria were analyzed using protein microarrays (Protoplex Immune Response Assay, ThermoFisher). A variable selection algorithm was applied to identify the smallest set of AAbs showing the best performance to classify malaria and bacteremia patients. The selection procedure identified 8 AAbs of which IFNGR2 and FBXW5 were selected in repeated model run. The classification error was 22%, which was mainly due to non-Typhi Salmonella (NTS) diagnoses being misclassified as malaria. Likewise, a cluster analysis grouped patients with NTS and malaria together, but separated malaria from non-NTS infections. Both current and recent malaria are a risk factor for NTS, therefore, a better understanding about the function of AAb in disease-specific immune responses is required in order to support their application for diagnostic purposes.

## Introduction

Differentiating malaria from alternative causes of febrile illnesses, in particular invasive bloodstream infections, is complex because of unspecific and overlapping disease symptoms. Reliable rapid diagnostic tests for malaria are available and in use^1^. However, due to semi-immunity, asymptomatic parasitaemia is common in holo-endemic malaria regions, which impedes accurate diagnoses in the presence of co-infecting pathogens. Thus, a positive malaria rapid diagnostic test result does not rule out other concomitant febrile diseases. Recent studies on causes of fever in Ghana and Tanzania showed that half of the children with parasitaemia revealed other diagnoses apart from malaria^2,3^. To address this challenge, the WHO recommends a combination treatment with broad-spectrum antibiotics and antimalarials for all cases of severe malaria in endemic settings, leading to high antimicrobial drug usage^4^. A diagnostic test, which is able to distinguish between malaria, bacteremia or a co-infection would facilitate empiric treatment decisions without prescribing unnecessary antimicrobial medication.

It has been shown that malarial infections lead to a range of characteristic immune responses, including hypergammaglobulinemia, polyclonal B cell activation and an increase in autoantibody (AAb) production^5^. It was shown that healthy individuals living in malaria hot-spots possess higher AAb levels than healthy individuals from malaria-free zones within the same country^6–8^. While AAbs are used for diagnosing several chronic inflammatory diseases^9–11^, little is known about their diagnostic potential in malaria, bacteremia or their use in the differential diagnoses of malaria from other infectious diseases.

The aim of the analysis is to identify the smallest set of AAbs capable to classify children with malaria or bacterial bloodstream infection. Using protein microarrays spotted with more than 9000 recombinant human proteins, we screened the serum of children infected with *Plasmodium falciparum* or with bacterial bloodstream infections.

## Results

Patients with bacteremia, malaria and controls were age and sex matched with a median age of 2 years (IQR 1–3) and 49% (n = 37) females in the study group. The majority of study participants were sampled during the rainy season (controls: 10 [100%], malaria: 30 [86%] and bacteremia 28 [93%]). The median parasite count in malaria patients was 181,670/µl (IQR 32,692–324,725) and the most frequently identified pathogen in the bacteremia group was NTS (n = 13; 43%) (Table 1).

**Table 1:** Characteristics of study participants stratified by study group.

Characteristics (statistics)	Bacteremia (30)	Malaria (35)	Control (10)
Age in years [median (IQR)]	2 (1–3)	2 (1–3)	1 (1–3)
Female [n (%)]	15 (50)	17 (49)	5 (50)
Parasite count/µl [median (IQR)]	NA	181,670 (32,692–324,725)	NA
**Bacterial isolate [n (%)]**			
Non-typhi *Salmonella* (NTS)	13 (43)	NA	NA
*Salmonella* Typhi	7 (23)	NA	NA
*Streptococcus pneumoniae*	5 (17)	NA	NA
*Staphylococcus aureus*	3 (10)	NA	NA
*Acinetobacter* spp.	1 (3)	NA	NA
*Campylobacter* spp.	1 (3)	NA	NA

*IQR* interquartile range, *n* sample size, *NA* not applicable.

Of the initial 9345 proteins on the array, 439 (5%) were excluded from the analysis because of elevated negative control values. Furthermore, 291 (3%) proteins were excluded because of batch effects, which left 8615 (92%) for further analyses. The distribution of AAbs ordered by their respective negative control values are shown in the Supplemental Fig. S1.

The proportion of AAbs per study participant with signal measurements above the respective AAb-median was assessed for controls, bacteremia patients and malaria patients. These values allow a relative quantification of high measurements in fluorescence intensities across disease groups (Supplemental Fig. S2). The median proportion of strong signals per observation was highest in malaria patients (71%; IQR 35–92), compared to bacteremia patients (40%; IQR 16–70) and controls (10%; IQR 5–16), which suggests higher fluorescence signals within the malaria group. There was no association between signal intensity and parasitaemia. However, among the bacteremia group, patients with NTS infections revealed a larger proportion of strong fluorescent signals compared to other bacterial species. Due to the low sample size, no further statistical analyses were applied.

### AAb selection by random forest

**Figure 1:**
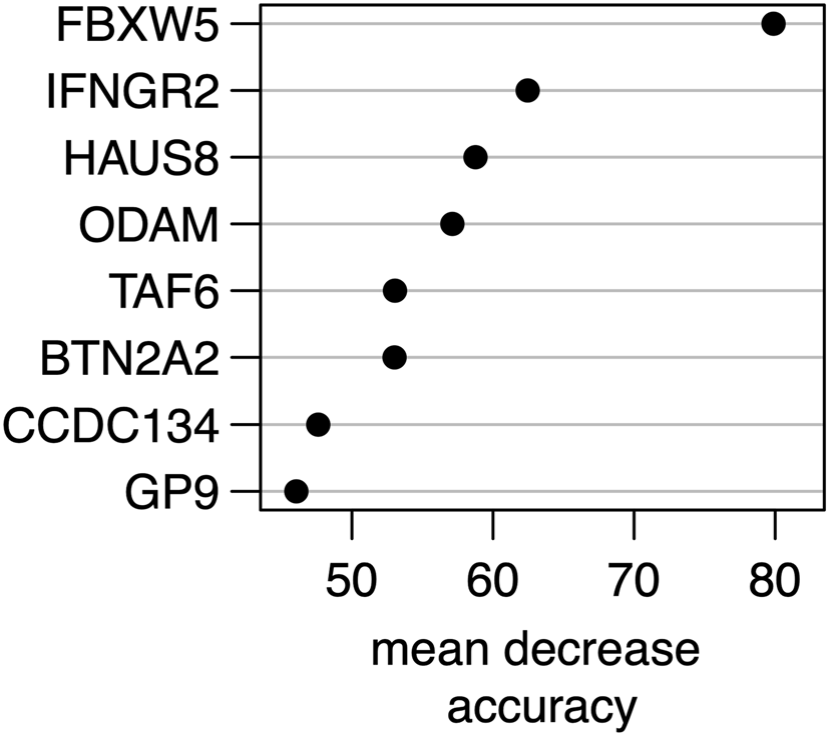
Variable importance of the eight autoantibodies based on the selected random forest model. Identifiers: *FBXW5* F-box-containing protein 5, *IFNGR2* Interferongamma receptor 2, *HAUS8* 8-Subunit Human Augmin Complex, *ODAM* Odontogenic, ameloblast associated, *TAF6* Transcription initiation factor TFIID subunit 6, *BT*N2A2 Butyrophilin subfamily 2 member A2, *CCDC134* Coiled-coil domain containing 134, *GP9* Glycoprotein IX (platelet).

Repeated random forest models were fitted to select the smallest set of AAbs with the best predictive accuracy. First, a random forest with all AAbs was calculated. Iteratively, 20% of markers with the lowest variable importance were removed and the models were recalculated. The full random forest model had a classification error of 42% and the median error over all iterative models was 31% (IQR 26–32). The smallest classification error rate of 22% was observed in the 32nd model fitted with 8 AAbs. Selected AAbs are described in Table 2 and their variable importance is shown in Fig. 1. To evaluate the performance of the selected model, patient’s disease-classes were predicted. The prediction error in the malaria group was 14% and, by far, lower compared to the bacteremia group (30%). Subdividing the bacteremia group into NTS and non-NTS isolates (i.e. all other bacterial species) showed that 8 (62%) of the NTS, but only 1 (6%) of the non-NTS patients were misclassified as malaria. Hence, the lower prediction accuracy in the bacteremia group was mainly due to NTS patients’ AAb profiles causing a misclassification as malaria.

**Table 2:** Autoantibodies selected by the random forest algorithm.

Antigen	UniProt^12^	Function	Category	Location
Butyrophilin subfamily 2 member A2 (BTN2A2)	Q8WVV5	Type 1 membrane protein, belongs to immunoglobulin superfamily, structurally related to family of T cell regulators (B7 family) (PMID: 23000944)	Immune response, signaling	Extracellular
Coiled-coil domain containing 134 (CCDC134)	Q9H6E4	Secretory protein, role in transcriptional regulation and MAPK signal transduction through Raf-1/MEK/ERK and JNK/SAPK pathways (PMID: 18087676)	Signaling	Intracellular, secreted
F-box -containing protein 5 (FBXW5)	Q969U6	Substrate recognition component of E3 ubiquitin-protein ligase complex (PMID: 10,531,035, 19,232,515)	Protein modification, signaling	Intracellular
Glycoprotein IX (platelet) (GP9)	P14770	Platelet surface glycoprotein, single-pass type I, part of receptor complex for von Willebrand factor (VWF) (PMID: 15,381,249)	Hemostasis, signaling	Extracellular
Interferon gamma receptor 2 (IFNGR2)	P38484	Single-pass type 1 membrane protein, forms IFN-γ receptor (together with IFNGR1), signal transduction in transcription regulation (PMID: 7673114)	Immune response, signaling	Extracellular
Odontogenic, ameloblast associated (ODAM)	A1E959	Tooth-associated epithelia protein, cancer related (PMID: 25911094)	Inflammation	Intracellular, secreted
8-Subunit Human Augmin Complex (HAUS8)	Q9BT25	Protein complex required for mitotic spindle assembly and centrosome integrity (PMID: 19427217, 19369198)	Structure, cell cycle regulation	Intracellular
Transcription initiation factor TFIID subunit 6 (TAF6)	P49848	Component of transcription factor IID complex (PMID: 15601843)	Signaling	Intracellular

**Figure 2:**
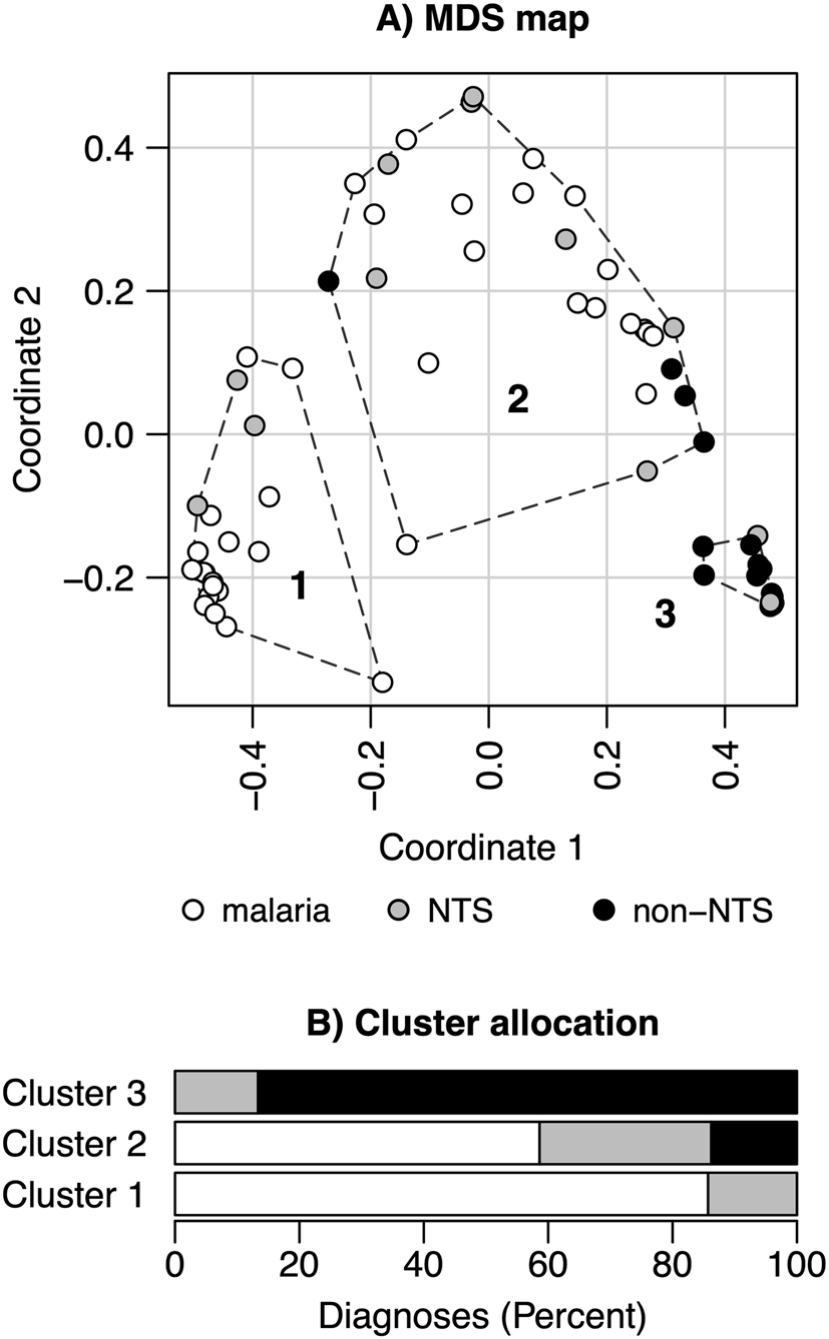
Cluster analysis. (**A**) Multidimensional scaling (MDS) map summarizing patient’s proximity in the final random forest model. Clusters are numbered and indicated by dashed lines. (**B**) The bars show the proportion of diagnoses allocated to the three clusters.

The proximity analysis highlighted the relatedness among malaria and NTS patients. Three patient-clusters were established from random forest’s proximity data, which are shown in the multidimensional scaling (MDS) map in Fig. 2A. The distribution of diagnoses within the clusters is displayed as bar charts in Fig. 2B. The first cluster primarily contained malaria patients (n = 18; 86%) along with 3 (14%) NTS patients. The median parasite density in malaria patients was 257,386/µl (IQR 61,266–399,546). Also, the second cluster was dominated by malaria patients (n = 17; 59%), with 8 (28%) NTS and 4 (14%) non-NTS patients. Compared to cluster 1, the median parasite count of malaria patients was lower (median = 106,470/µl, IQR 22,143–262,150). Within the third cluster the majority of non-NTS patients (n = 3; 87%) were grouped, along with only 2 (13%) NTS and no malaria cases.

### Autoantibody induction levels

**Figure 3:**
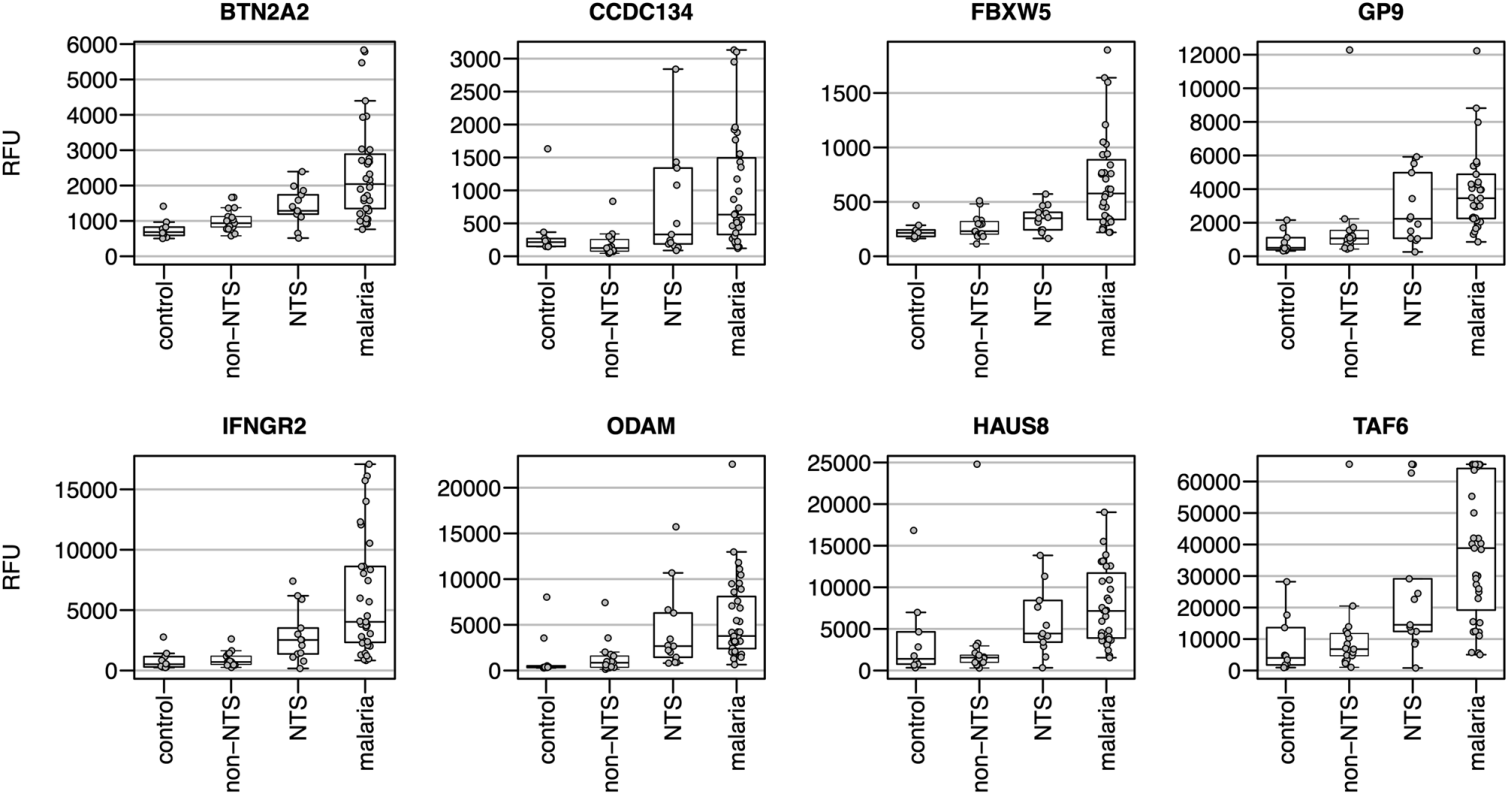
AAb induction levels. The induction levels of the eight selected autoantibodies are shown for controls, non-NTS, NTS and malaria patients. Identifiers are explained in Table 1. *RFU* relative fluorescence unit, *NTS* non-Typhi Salmonella, *non-NTS* bacterial species other than NTS.

Induction levels of the eight AAbs selected by random forest are shown in Fig. 3. For most markers, the median induction levels were lowest in controls, followed by non-NTS and NTS cases, and were highest in patients with malaria. Only in CCDC134 the control group showed a median induction level above cases with other bacterial species than NTS (non-NTS). In all AAb, the malaria group had the highest variability in the measured induction levels compared to the other groups. However, the individual parasite count did not correlate with AAb induction levels. Categorization of selected AAbs according to the predicted function of their antigen (as outlined in Table 2) showed that 6 of 8 are in some way involved in signaling (BTN2A2, CCDC134, FBXW5, GP9, IFNGR2, and AF6), while the other two are involved in inflammation (ODAM) and the cell cycle (HAUS8). Antigen localization showed that five of the eight AAb targets are intracellular (FBXW5, HAUS8, TAF6), whereby two are also secreted into the extracellular environment (CCDC134, ODAM), and three targets are extracellular (BTN2A2, GP9, IFNGR2).

### Robustness of study results

**Figure 4:**
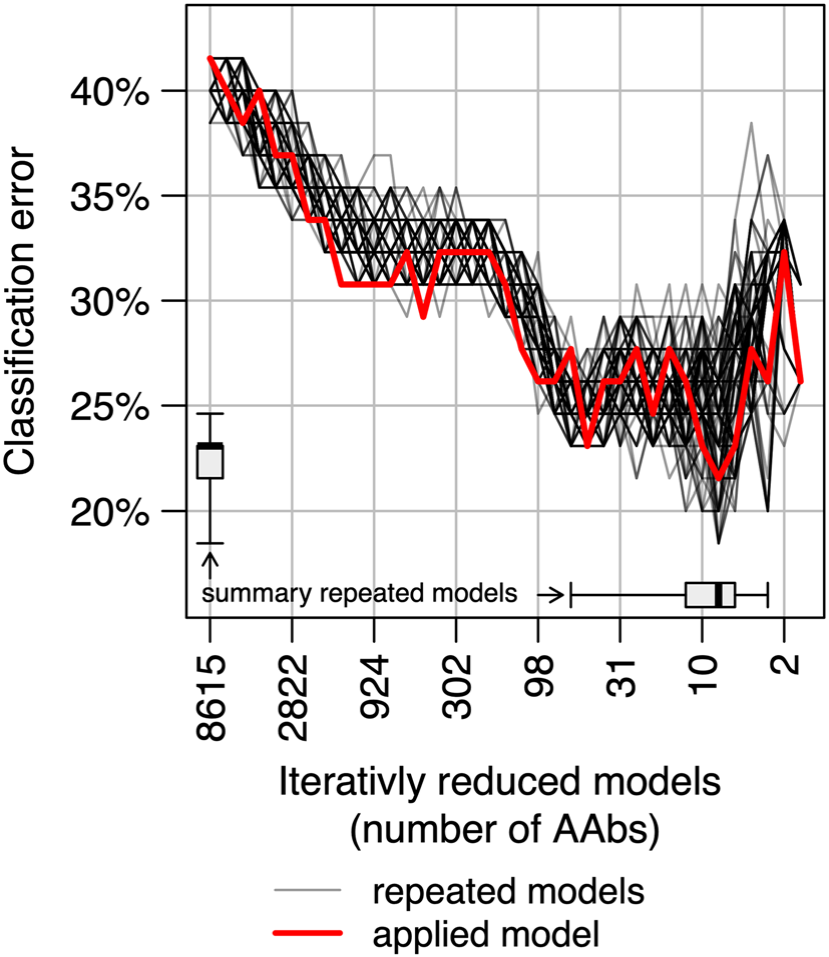
Classification error. The plot displays the robustness of the variable selection models by summarizing the distribution of classification errors over 100 marker selection algorithms. The x-axis shows the number of markers in a model and the y-axis the classification error of the respective random forest models. The change in classification error within the repeated marker selection algorithms are shown by the gray lines. The applied model is displayed by the red line. The boxplots show summary statistics about the number of AAb in finally selected models (y-axis) and the lowest classification errors (y-axis) in the repeated models.

**Figure 5:**
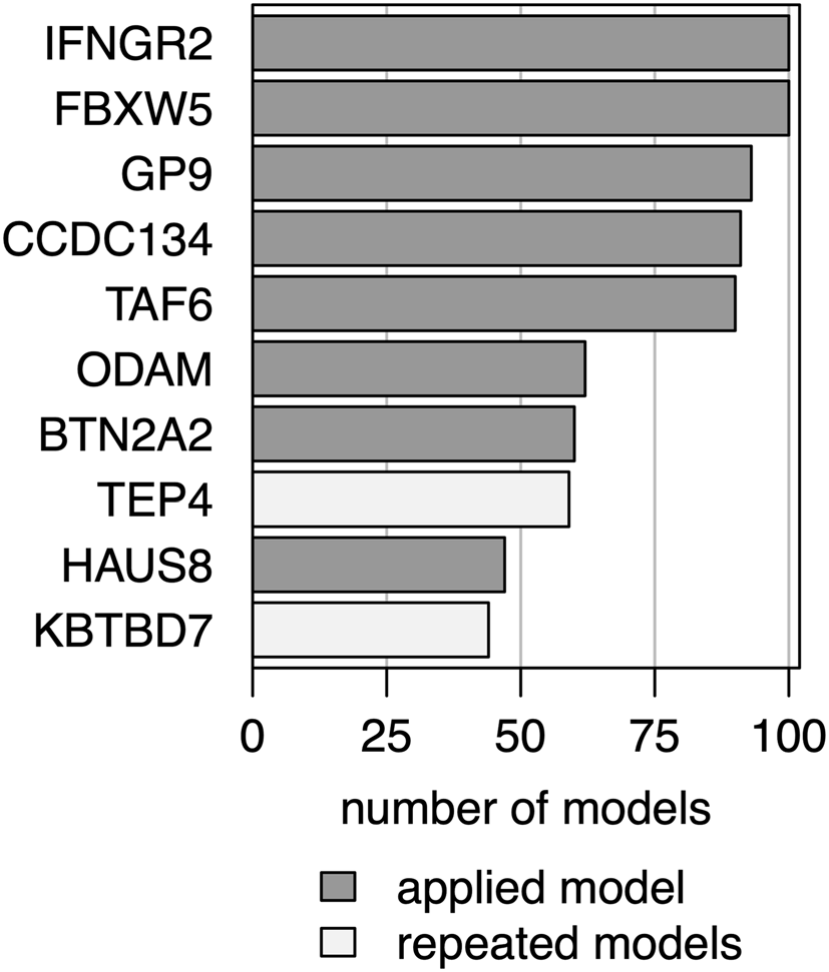
AAb selection model. Number of times where AAbs were selected in a repeated model. Markers selected by the applied model are colored dark gray and markers only selected in the repeated models are colored in white. Identifiers of AAbs not listed in Table 2: TEP4 (Transducin-like enhancer protein 4), KBTBD7 (kelch repeat and BTB (POZ) domain containing 7).

Robustness of the random forest selection approach was tested by repeating the selection algorithm 100 times. The median classification-error rate in the 100 selected models was 23% (IQR 22%–23%), compared to 22% in the applied model. The smallest classification-error was most often measured in a model containing eight AAbs (n = 29, 29%), which is in line with the applied model. The smallest marker-set selected contained 3 markers and the largest 62. Figure 4 summarizes the classification-error distribution over the 100 algorithm runs, while the finally applied model selection algorithm is printed in red. In total, 85 different AAbs were identified by repeating the selection algorithm. Figure 5 shows the 10 AAbs most often selected by the repeated models. All eight markers identified by the applied algorithm belong to these 10 markers. Notably, IFNGR2 and FBXW5 were selected in all 100 model runs.

## Discussion

We established AAb profiles using protein microarrays for children with malaria or bacterial blood stream infections in order to identify disease specific induction patterns. These patterns were then tested for their ability to predict disease status, in order to identify AAb candidates capable to detect the underlying pathogen responsible for the acute infection. In general, most individuals have low autoantibody titers, while high levels are associated with certain autoimmune diseases. Antinuclear antibodies (ANAs) for example are antibodies that recognize and bind to structures of the nucleus, like e.g. double-stranded DNA^13^. Multiple studies over the years observed a high incidence of ANA in healthy individuals living in malaria-endemic areas of Africa, but a lack thereof in people from malaria-free areas within the same country^6,14,15^. The occurrence of these ANAs together with high serum levels of anti-malaria antibodies led investigators to believe that this phenomenon might be malaria-induced^7,8^. Over the years, technological advances in biomedical research, broader autoantigen panels and high-throughput experiments in array format have now provided evidence for a greater number of AAbs to be associated with malaria^16–21^.

The present analysis demonstrates an increased number of recognized self-antigens by AAbs, a higher signal intensity and response variability in patients with malaria compared to those with bacterial bloodstream infections. Especially children diagnosed with non-NTS isolates could be well separated from malaria patients. Our results allude to a resemblance between AAb induction profiles between malaria and NTS patients. Malaria and invasive NTS infections have a similar age distribution and their prevalence overlap geographically^22^. Previous studies have demonstrated an increased risk for malaria patients to have a concomitant or subsequent invasive NTS infection^23,24^. Inclusion criteria did not cover a history of fever dating back further than 48 h prior to enrolment in the study. Therefore, it is possible that the NTS group, or a fraction thereof, rather represents AAb profiles of a recent malaria episode instead of a current NTS infection.

While it is accepted that infections may trigger AAb production, functional consequences and molecular mechanisms leading to autoimmunity remain unclear. Molecular mimicry, epitope spreading, bystander activation, polyclonal B cell activation are a few hypothesized mechanisms that could be involved^25,26^. Recently, tumor-associated AAbs have expanded the field of cancer immunodiagnostics. In that context, it was noted that AAbs could be viewed as imprints of the ongoing immune response in the body. Even though only a few AAbs have been shown to be disease associated, different profiles have been linked with different diseases and could be useful as diagnostic markers apart from autoimmune diseases^27,28^.

IFN-γ is a cytokine of the innate and adaptive immune system and has important functions in a number of immune-related processes. While IFN-γ plays a central role in controlling blood and liver stage malaria in humans and mice^29,30^, it has also been shown to have an aggravating effect for the course of infection in mice^31,32^. Transcriptional profiling of tissue cultures showed that the absence of IFN-γ receptor signaling led to an increase in parasite load in *Toxoplasma gondii* infection^33^ but contributed to experimental cerebral malaria pathogenesis in mice^31,32,34^.

Mendelian susceptibility to mycobacterial disease (MSMD) is a rare inherited condition where mutations in autosomal genes in pathways involving interferon-γ (as well as interleukin-12, and tumor necrosis factor α, TNF-α) cause a primary immunodeficiency and predisposes individuals to a wide range of infections, including NTS^35,36^. Autoantibodies against cytokines were found in the course of MSMD research, where anti-IFN-γ autoantibodies seemed to mimic inborn errors and interfere with IFN-γ signaling and correlate with disseminated opportunistic infections, including Salmonella^37^.

The other selected candidate was FBXW5, a member of the family of F-box proteins that make up one of the four subunits of the ubiquitin protein ligase complex (also called SCF complex), which in turn plays a role in protein degradation^38^. It contributes to the substrate specificity of the SCF complex and is also essential for its regulating during the cell cycle^39^. Despite its basic molecular function, nothing much is known with respect to its role in infection. It will be crucial to validate our findings and further elucidate the (diagnostic) role of FBXW5 in malaria or infections in general.

The large number of AAbs, in combination with the limited number of patient samples, is likely to produce chance findings. Thus, the current analysis is of exploratory nature and does not allow to determine the predictive power of particular AAbs under study or to select combinations of markers to be applied in a clinical setting. The eight candidates identified in this analysis need to be confirmed with well-characterized patient samples from an independent study in order to determine their diagnostic potential. A general limitation of serological testing in acute infections is the fact that disease-specific antibodies need several days after the onset of fever before being detectable in peripheral blood^40^. In addition, antibody decay is lower than parasite clearance rates. Chronic exposure in malaria-endemic regions leads to a constant background seroprevalence, which limits serological tests in their sensitivity and specificity for diagnostics in acute infections^41,42^. Further, the lack to distinguish patients with NTS from patients with malaria is a serious limitation with negative consequences for differential diagnostics in malaria endemic settings.

The current study showed different AAb induction profiles in children diagnosed with malaria or bacteremia, whereby bacteremia cases infected with bacterial species other than NTS showed a distinct AAb pattern compared to malaria patients. In order to improve differentiation between malaria and NTS, better defined patient groups, e.g. with data about recent malaria infections, would be necessary. Taken together, this study identified candidates, which should be subject to further investigation. A validation of our prediction models is a promising next step in the development towards a new pathogen specific rapid diagnostic test.

## Materials and methods

### Ethics statement and informed consent

The study was performed in accordance with the relevant Ghanaian and German guidelines and regulations, and with the Declaration of Helsinki. Ethical approval for the study had been obtained from the Committee on Human Research, Publications, and Ethics, School of Medical Science, Kwame Nkrumah University of Science and Technology (KNUST), Kumasi, Ghana (Reference number: CHRPE/101/09) and the Institutional Review Board (IRB) of the International Vaccine Institute. Written inform consent was obtained from the participants. All parents or legal guardian were informed about the study’s purpose and procedures, and provided written informed consent prior to enrollment.

### Study area, study group

Recruitment took place between February 2010 and May 2012 in the framework of a pediatric fever study^43^. Serum samples from 65 children (≤ 5 years of age) admitted to the children’s ward of the Agogo Presbyterian Hospital (APH) in Ghana with fever (≥ 38 °C) were used for this study. Thirty-five children had *P. falciparum* parasitaemia and a negative blood culture, while 30 children had bacteremia with a negative malaria slide. As control group, asymptomatic children without fever and no signs of infection were selected and sampled in the frame of another fever study^2^ at vaccination clinics in the surroundings of the APH. Data from control children were displayed as reference values, however not used in the classification analysis, because the low sample size does not allow to predict a third outcome group. Children in all groups were matched by age and sex.

APH is a district hospital with 250 beds, situated in the Asante Akim North Municipality in Ghana. The region has a tropical climate and is highly endemic with *P. falciparum*.

### Microbiological analysis

Blood cultures were performed with pediatric blood culture bottles (Becton Dickinson (BD) BACTEC Peds Plus/F) using an automated BACTEC 9050 Blood Culture System (BD, Franklin Lakes, NJ USA) as described elsewhere^43^. Bacterial identification was achieved biochemically with API tests (bioMérieux, Marcy L’Etoile, France) and *Salmonella* isolates were serotyped following the White-Kauffmann-Le Minor scheme. For data analysis bacteremia patients were classified as non-typhoid *Salmonella* (NTS; i.e., patients with invasive NTS) and non-NTS (i.e., causative agents of bacterial bloodstream infections other than NTS). Two independent slide readers conducted malaria microscopy on Giemsa stained thick and thin smears. In case of discrepancies in parasite counts, a third decisive reading by an additional reader was performed.

In order to investigate the autoantibody profile of the serum samples, protein microarrays were performed (ProtoPlex Immune Response Assay, ThermoFisher). Herein, 9345 human proteins were expressed as GST-tagged proteins in insect cells and purified under native conditions to maintain their native conformations and posttranslational modifications^44^. Purified proteins were spotted on nitrocellulose-coated glass slides. These were blocked, washed and probed with a 1:500 dilution of selected human serum samples. A negative control assay incubated with buffer instead of serum served as a negative control to exclude non-specific interactions from the analysis. Alexa Fluor 647-conjugated goat anti-human IgG antibodies were used for detection and array signals were read using a Tecan PowerScanner fluorescent microarray scanner. The resulting signals were equated with AAb profiles. Relative Fluorescence Units (RFU) for each spot on the array was determined using GenePix 7 software (Molecular Devices LLC, CA, USA) and signals were analyzed as described below.

### Data analysis and statistical methods

Pre-processing steps were applied to prepare data for analysis. Background correction was conducted using the normal-exponential convolution method. Negative control values, which capture the fluorescence intensity of a marker without serum, were evaluated. Markers with negative control values above the 95^th^-percentile of the overall negative control distribution were deemed unspecific and excluded. Markers were analyzed in two batches. Antigens that were not recognized by any serum samples in either of these batches were removed from the dataset. No further batch effects, like varying induction intensity levels among batches, were detected.

Random forest was applied to identify the smallest set of AAbs showing the best performance to classify malaria or bacteremia patients in our dataset. Random forest is an ensemble learning method based on multiple classification and regression trees. Classification trees were used in our analysis, since we categorized observations according to their respective diagnoses. Each tree within a forest is built using a random subset of observations, and a random subset of variables are applied to group observations at each split. Using this approach, low-biased and low correlated individual trees are established, over which the final result is averaged. Classification error is estimated internally each time a tree is constructed by predicting the so called out-of-bag (OOB) observations, which were not considered while constructing a tree. This prevents overfitting since internally training and test datasets are utilized. Random forest calculates a variable importance measure for each variable, which is the increase in classification error if values of a variable would be permuted randomly^45^. Random forest shows very good classification performance in high dimensional data, i.e. when the number of variables exceeds the number of observations, and when most predictor variables are noise^46^.

To select a set of AAbs with best classification performance we followed the selection approach as described by Díaz-Uriarte et al*.*^47^. In a first step we fitted a full random forest considering all AAbs and we kept the variable importance data. Iteratively, the 20% of AAbs with the smallest variable importance were removed and the remaining variables were used to re-run random forest. Variable importance was not recalculated for predictor selection since this would cause overfitting. Finally, we selected the AAb set with the smallest OOB-classification error from the model^47^.

The final model, fitted with the selected set of AAbs, was evaluated to identify clustering among study participants. In random forest, the proximity of each observation-pair is computed based on the frequency cases occupy the same terminal node within trees, assuming that similar observations are likely to share a terminal node. The dimensions of the proximity matrix were reduced to two coordinates by multidimensional scaling (MDS) to visualize similarities between observations with a scatter plot. In order to partition patients into clusters, we applied the k- medoids algorithm on the proximity matrix.

Random Forests were constructed with the following parameter setting: default number of variables considered per split (i.e., square root of the number of AAbs), each forest contained 20,000 trees and the reciprocal frequency of malaria and bacteremia patients was used to weight classes (i.e., malaria = 30, bacteremia = 35) to account for imbalanced outcome groups. Each tree was unpruned and could grow fully to the largest extend possible. Random predictor and observation allocation in random forest influence model results and consequently the set of markers selected by the algorithm. Thus, the robustness of the selection procedure was evaluated by repeating the marker selection algorithm 100-times.

All analyses were performed with *R* (version 3.6.1) using the packages *PAA* (version 1.10) for AAb pre-procession, *randomForest* (version 4.6–14) to fit random forest models and *cluster* (2.10) to partitioning patient clusters*.*

## Supplementary information

Supplementary Information.

## Data Availability

All data generated or analyzed during this study are included in this published article (and its Supplementary Information files).
