# Supplementary material for: Classification of invasive bloodstream infections and Plasmodium falciparum malaria using autoantibodies as biomarkers
Source: Sci Rep. 2020 Dec 3;10:21168. doi: 10.1038/s41598-020-78155-y (PMC7712777; doi:10.1038/s41598-020-78155-y)
Supplement: Supplementary file 1 — Supplementary Information. [file 41598_2020_78155_MOESM1_ESM.pdf]

Classification of invasive bloodstream infections and *Plasmodium falciparum* malaria using autoantibodies as biomarkers

Ralf Krumkamp[a,b¶], Nicole Sunaina Struck[a,b¶*], Eva Lorenz[a,b], Marlow Zimmermann[a,b], Kennedy Gyau Boahen[c], Nimako Sarpong[c], Ellis Owusu-Dabo[d], Gi Deok Pak[e], Hyon Jin Jeon[e], Florian Marks[e,f], Thomas Jacobs[g], Jürgen May[a,b,h&], Daniel Eibach[a,b&]

[a]Department of Infectious Disease Epidemiology, Bernhard Nocht Institute for Tropical Medicine, Hamburg, Germany

[b]German Centre for Infection Research (DZIF), Hamburg-Lübeck-Borstel-Riems, Germany

[c]Department of Infectious Disease Epidemiology, Kumasi Centre for Collaborative Research in Tropical Medicine (KCCR), Kumasi, Ghana

[d]School of Public Health, Kwame Nkrumah University of Science and Technology (KNUST), Kumasi, Ghana

[e]Epidemiology Unit, International Vaccine Institute (IVI), Seoul, Republic of Korea

[f]The Department of Medicine, the University of Cambridge, Cambridge, United Kingdom

[g]Research Group Protozoa Immunology, Bernhard Nocht Institute for Tropical Medicine, Hamburg, Germany

[h]First Medical Clinic and Polyclinic, University Medical Center Hamburg-Eppendorf, Hamburg, Germany

¶These authors contributed equally to this work.

&These authors also contributed equally to this work.

*Corresponding author:

Bernhard-Nocht-Institute for Tropical Medicine

Bernhard Nocht Str. 74; 20359 Hamburg, Germany

Tel.: +49 40 42818 639

Email: struck@bnitm.de

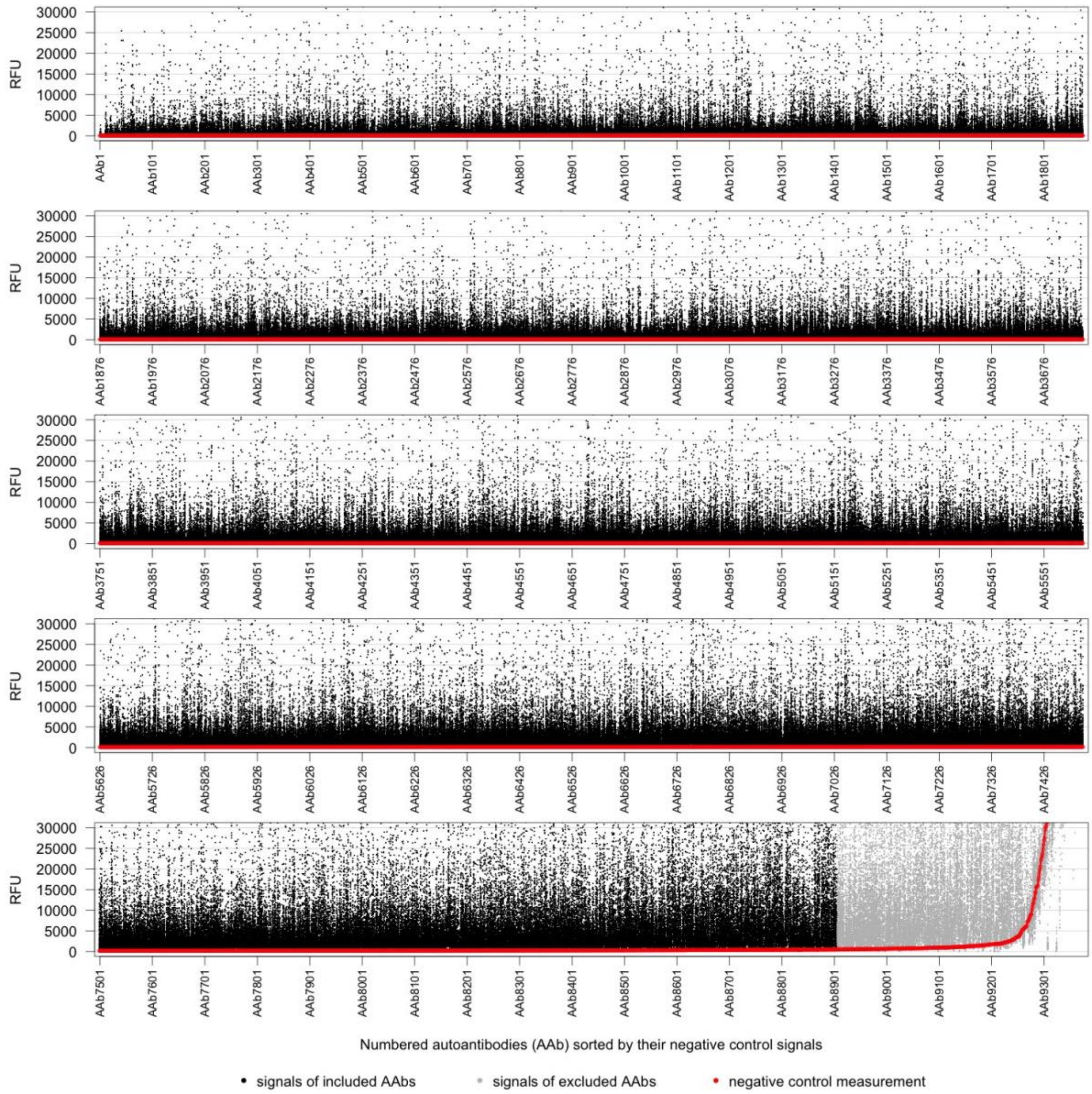

Figure S1: Negative Control. Induction signals of all AAbs ordered by negative control signals. AAbs plotted in grey are excluded because of a negative control value above the 95th-percentile, black AAbs are considered for analysis, and negative control values are shown in red.

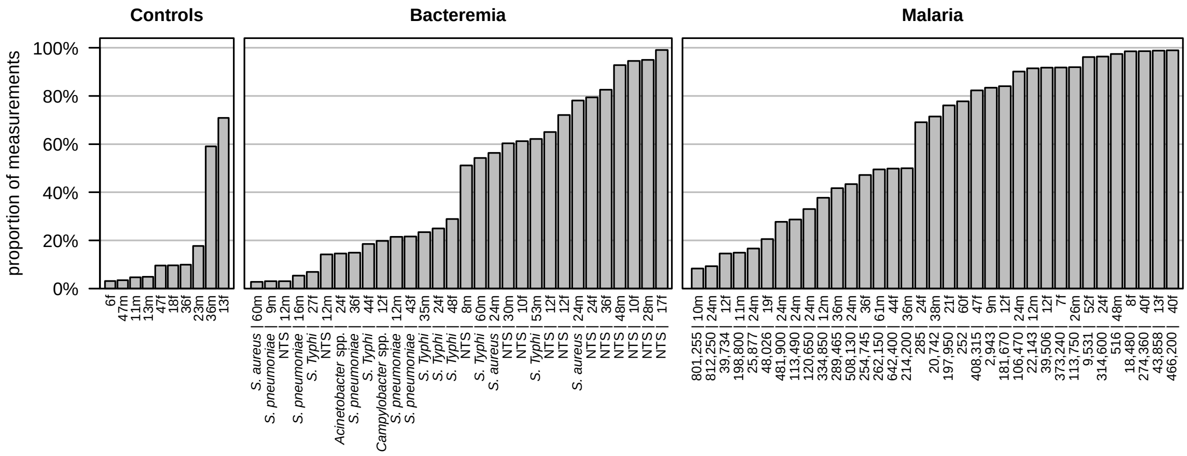

Figure S2: Proportion of pre-selected autoantibodies with signal measurements above an AAb's median for each child. The x-axis accounts for the diagnosed bacterial isolate in the bacteremia group or the malaria parasite count per μl in the malaria group, respectively, along with the patient's age in months and sex (f, female; m, male). The y-axis displays the proportion of AAb signals above the respective AAb-median by study participants.
